# Anomalous Experiences, Trauma, and Symbolization Processes at the Frontiers between Psychoanalysis and Cognitive Neurosciences

**DOI:** 10.3389/fpsyg.2015.01926

**Published:** 2015-12-21

**Authors:** Thomas Rabeyron, Tianna Loose

**Affiliations:** ^1^Department of Psychology, University of NantesNantes, France; ^2^Department of Psychology, University of EdinburghEdinburgh, UK; ^3^Department of Psychology, University of Québec in MontrealMontreal, QC, Canada

**Keywords:** anomalous experiences, paranormal, trauma, hallucinations, altered states of consciousness, psychic permeability, symbolization

## Abstract

Anomalous or exceptional experiences are uncommon experiences which are usually interpreted as being paranormal by those who report them. These experiences have long remained difficult to explain, but current progress in cognitive neuroscience and psychoanalysis sheds light on the contexts in which they emerge, as well as on their underlying processes. Following a brief description of the different types of anomalous experiences, we underline how they can be better understood at the frontiers between psychoanalysis and cognitive neurosciences. In this regard, three main lines of research are discussed and illustrated, alongside clinical cases which come from a clinical service specializing in anomalous experiences. First, we study the links between anomalous experiences and hallucinatory processes, by showing that anomalous experiences frequently occur as a specific reaction to negative life events, in which case they mainly take the form of non-pathological hallucinations. Next, we propose to analyze these experiences from the perspective of their traumatic aspects and the altered states of consciousness they often imply. Finally, these experiences are considered to be the consequence of a hypersensitivity that can be linked to an increase in psychic permeability. In conclusion, these different processes lead us to consider anomalous experiences as primary forms of symbolization and transformation of the subjective experience, especially during, or after traumatic situations.

## The psychology of anomalous experiences

The origin of the scientific and clinical understanding of anomalous experiences dates back to the end of the nineteenth century, and is based particularly on work conducted by scholars and members of the *Society for Psychical Research*, in Cambridge, the *American Society for Psychical Research*, in Boston, and the *Institut Métapsychique International* in Paris (Méheust, 1999). At this time, philosophers, doctors, and psychologists such as James (1902), Myers (1903), Richet (1923), Bergson (1932), and Freud (Freud, 1933; Devereux, 1953; Massicotte, 2014) were attempting to understand strange experiences that seemed to resist scientific explanation, but that were potentially important for the understanding of the human mind.

Members of the *Society for Psychical Research* (Gurney et al., 1886) published the conclusions of one of their largest studies in *Phantasms of the Living.* Seventeen thousand people responded to the question “Have you ever had a realistic impression of seeing, hearing or being touched by a living being or an inanimate object that didn't seem to have an external cause when you were completely awake?.” One in every 10 people responded affirmatively to this question, and recent studies suggest that this proportion remains approximately the same in the present day (Johns et al., 2004). However, the epistemological and sociological status of these experiences, along with their complexity, and their unthinkable nature at this time (Méheust, 1999), led them to become progressively marginalized in mainstream psychology during the twentieth century (Le Maléfan, 2000; Evrard, 2014).

As we will propose in this paper, current progress in cognitive neurosciences and psychoanalysis allows us to better understand these human experiences, which were difficult to integrate into scientific models until recently. The last few years have seen many books and articles published on the topic of what are now called “anomalous experiences,” mainly from the point of view of cognitive neurosciences (Brugger and Mohr, 2008; Krippner and Friedman, 2010), psychoanalysis (Eschel, 2006; Brottman, 2011; Si Ahmed, 2014), clinical psychology (Kramer et al., 2012), and anomalistic psychology (Holt et al., 2012; French and Stone, 2013; Cardeña et al., 2014). Several definitions of anomalous, exceptional or “subjective paranormal experiences” (Neppe, 1980) have been proposed. They are defined by Cardeña et al. (2014) as “uncommon experiences (e.g., synesthesia), or those that, although they may be experienced by a significant number of persons, are believed to deviate from ordinary experience or from the usually accepted explanations of reality according to Western mainstream science” (p. 4). Metzinger (2003) defines them as “deviations from what might be referred to as ordinary experiences, i.e., experiences consistent with typical ‘reality models” that individuals develop to cope with their socio-cultural environment” (p. 16). Between 30 and 50% of the population report having had at least one such experience (Ross and Joshi, 1992; Evrard, 2013a) that involves the subject being confronted with an “anomaly” that usually elicits intense positive or negative emotions. In terms of demographics, these experiences are manifested amongst people of all ages, genders, education levels, and cultures (Evrard, 2013a). Moreover, even if there is a complicated relationship between anomalous experiences and psychopathology, they can't be reduced to purely psychopathological phenomena (Evrard, 2013a).

In taking a phenomenological approach, anomalous experiences can be classified into 10 categories (Rabeyron et al., 2010). Firstly, some of these experiences belong to a category involving an unusual “perceptive” interaction between a person and his environment. Thus, during (1) *psi perceptions*, the person believes he or she has been able to obtain information from another person (telepathy), at a distance (clairvoyance) or from the future (precognition) (2) In *Vision and apparition* experiences the presence of something or someone is perceived in a quasi-hallucinatory manner, and is often related to or considered to be a real event. (3) *Out of Body Experiences* (OBE) implicate a change in body awareness and lead to the feeling of being situated outside of one's body (McCreery and Claridge, 2002; Blanke and Dieguez, 2009). From a more “projective” perspective, some people believe they have a “paranormal” influence on their environment. This is the especially the case in (4) *subjective psychokinesis experiences*, which involve the perceived ability to interact mentally with objects, and (5) *magnetism or healing experiences*, which suppose inexplicable interactions between living systems (Schmidt et al., 2004). A third category concerns “encounter experiences” in which “another world” is usually reported. This includes (6) *Near Death Experiences* (NDE) (van Lommel et al., 2001; Mobbs and Watt, 2011), which occur especially after comas or clinical death, and which some people (after seeing, for example, a tunnel or deceased loved ones) interpret as being a journey in the after life (Parnia et al., 2014). A belief in life after death is also frequent in (7) *mediumistic experiences*, which correspond to the alleged ability to communicate with the deceased. In experiences of (8) *reincarnation*, the person, sometimes a child, believes that they have past life memories (Stevenson, 1967; McNally, 2012). (9) *Mystical experiences* are also in this encounter category, and relate to an intense and global feeling of having “become one” with God or the universe. Finally, maybe the most surprising of these experiences are (10) *abductions*, in which people are strongly convinced that they have been abducted by aliens (Clancy et al., 2002).

It is common for a single person to report having had a variety of anomalous experiences, especially after a first “inaugural” experience (Rabeyron, 2012). Accordingly, it seems appropriate to approach and conceptualize them as a whole, both from a clinical and a theoretical perspective. In this regard, anomalistic psychology tries to understand and explain these experiences (Holt et al., 2012; French and Stone, 2013), whereas the clinical psychology of anomalous experiences aims to develop therapeutic models in order to help people cope with these experiences when they are associated with mental suffering (Fach et al., 2013; Landolt et al., 2014). Psychotherapy can indeed be useful in order to re-establish the individual's well-being, especially in counseling services that are specifically dedicated to anomalous experiences (Belz-Merk, 2000). From this point of view, these experiences question how clinicians are supposed to deal with patients reporting unusual experiences that do not fit with the patient's or the therapist's model of reality, and that frequently engender an “ontological choc” (Mack, 1995). This question relates to a recent study in Holland conducted on 640 mental health professionals (Corbeau, 2004; Evrard, 2013a), in which half of those who responded reported having listened to narratives of anomalous experiences produced by their patients. Four out of five clinicians explained that they were lacking in the relevant information on this topic which would allow them to care for their patients appropriately. This topic also has more global ramifications concerning the way in which clinicians deal with hallucinatory and seemingly psychotic experiences, leading for example to the development of the hearing voice networks that help people cope with auditory hallucinations (Romme and Escher, 2000).

As we will propose in this paper, these experiences are best understood at the frontiers between different fields of knowledge, particularly the domains of neuroscience and psychoanalysis. Interestingly, Freud himself had a great interest concerning what was called “occultism,” and he published several papers dealing especially with the question of thought-transference (Freud, 1921, 1922, 1925, 1933), suggesting that it posed significant questions for psychoanalytic understandings of mental processes. While psychoanalysis and clinical psychology contribute to a detailed understanding of the patient's subjective experience, cognitive neurosciences provide the conceptual framework necessary for testing and exploring hypotheses generated in clinical settings. As in the more global project of neuropsychoanalysis (Kandel, 1999; Bazan, 2011; Solms and Turnbull, 2011), the double stance offered by psychoanalysis and cognitive neurosciences allows us to better understand human psychic functioning (Northoff, 2012; Ruby, 2013). Given the complexity of anomalous experiences, their subtle and varying relationship with mental disorders, and their consequences, which can have both positive and negative effects on mental health, their specificity is more easily understood at the border between psychoanalysis and neurosciences. Moreover, the combination of these two domains allows us to reach levels of complexity at which these experiences became better understood, and avoids the risk of reductionism, which might occur if we considered only some aspects of these experiences.

If neurosciences and psychoanalysis can be used to better understand anomalous experiences, these experiences can, on the opposite, be relevant to better understand neurosciences and psychoanalysis, given that it is often at the margins that new discoveries on more usual experiences are made. This parallels the way in which psychopathology sheds light on normal functioning. For example, a great deal of interest has recently been focused on Out of Body Experiences (OBE), because they seem to foster our understanding of the relationship between embodiment processes and the self (Blanke et al., 2002, 2004). Virtual reality set-ups have been developed in order to artificially produce the same kind of kinesthetic illusion (Ehrsson, 2007), and these experimental designs have also improved our understanding of phantom limb pain (Ortiz-Catalan et al., 2014). Neurosciences help us to determine the neurological processes associated with these experiences, while psychoanalysis improves our understanding of these experiences from a subjective point of view. Indeed, the neurological basis of the OBE can hardly explain the spontaneous emergence of these experiences, their psychogenic origin or the way in which they fit into psychic reality as a whole. Recent research has also emerged that conceptualizes Near Death Experiences (van Lommel et al., 2001; Parnia et al., 2014) as a new paradigm that aims to study consciousness and memory processes during and after transitory clinical death (Thonnard et al., 2013; Charland-Verville et al., 2014). While neuroscience helps describe the neurological structures and mental functioning associated with NDE, psychoanalysis sheds light on the transformative processes following NDE (Greyson, 1983, 2007)^1^.

In this regard, our aim in this paper will be to expose several axes of research at the intersection between psychoanalysis and cognitive neurosciences, including hallucinatory processes, altered states of consciousness, psychic permeability and symbolization. We will also base our analysis on clinical cases^2^ coming from a counseling service specialized in anomalous experiences^3^ (Rabeyron et al., 2010).

## Anomalous experiences, hallucinations, and hallucinatory processes

People contacting the counseling service usually see, hear or feel things that do not seem, for them, to have an internal cause. They will interpret these anomalies concerning their perception of reality in many different ways, depending on the type of experience and their associated beliefs. They usually have a profound desire to share these experiences, even if they are afraid of being labeled as crazy or insane. This means that a lot of them avoid speaking about them to their general practitioner, hence staying out of the “psychiatry loop.”

A first approach would involve considering these experiences as hallucinatory processes in the general population. As previously mentioned, members of the SPR had identified a high prevalence of seemingly hallucinatory processes in the general population, and these results have since been confirmed by several studies showing that almost 10% of the population report having had vivid hallucinatory experiences (Johns et al., 2004). Some researchers have proposed that hallucinatory experiences could be the expression of a larger psychotic phenotype that does not automatically equate to a pathological expression (Strauss, 1969; van Os et al., 2000). For example, van Os et al. (2000) looked at the prevalence of experiences akin to psychosis amongst 7075 subjects from the general population. 4.2% of them had psychiatrist-rated evidence of delusions or hallucinations, and 17.5% reported experiences belonging to the register of psychosis even though they did not suffer from schizophrenia. Johns and van Os (2011) concluded that “there are normal individuals who experience hallucinations under no special circumstances, and surveys have shown that more people experience hallucinations that come into contact with medical or psychiatric services” (p. 1129). van Os et al. (2000) also note that the psychotic phenotype could be fifty times more prevalent than schizophrenia in the general population. van Os et al. (2009) remark more precisely that psychotic experiences could be experienced by almost 8% of the general population, in which 4% would develop psychotic symptoms and 3% psychotic disorders. The limits of the psychotic phenotype would thus not overlap with the limits of the clinical diagnostic of psychosis and the categorical approach of this disorder.

Those who report anomalous experiences could belong to this category of people who have hallucinations without clear expression of mental health suffering. It is then debatable to consider these hallucinatory processes as an early warning sign of psychotic disorders, given that the origin and implications of these hallucinatory processes are not agreed upon because of the complexity of the implicated factors. In this regard, results from cognitive neurosciences in this domain could be useful to better understand the nature of these experiences. Thus, the original definition of hallucinations proposed by Esquirol (1832)—the perception of an object in the absence of an object to perceive—seems to be outdated, and models have since been developed in order to understand hallucinations from neurobiological, affective and cognitive perspectives. Several models in cognitive neurosciences consider in particular hallucinations from the point of view of “reality monitoring,” which is a process that lies within the broader category of “source monitoring.” According to Bentall (1990), hallucinations should be considered as the consequence of a faulty categorization: the brain interprets an internal perception, representation or memory as coming from an external source instead of an internal one. Hallucinations would thus be the result of an alteration of the “judgment of reality” processes and this alteration could be influenced by a failure in the balance between top-down and bottom-up processes (Allen et al., 2008). Some authors have also proposed that a hyperactivity of resting state would lead the subject to an externalized perception of its internal experience (De Masi et al., 2015), while a hyperactivity of perceptive areas would be associated with a decrease in the superior function used for the judgment of reality. Hallucinations could then represent a kind of “psychic retreat” (Steiner, 1993; De Masi et al., 2015) whose aim would be to provoke a withdrawal from the world, allowing the subject to avoid anxiety (Delespaul et al., 2002). The subject is then “cut” from his relational and adaptive capabilities, given an antagonist relationship between hallucinations and attention on the external world (De Masi et al., 2015).

The role of environmental and emotional contexts is also crucial in the experience of hallucinations, and numerous environmental factors—such as, for example, urbanity or familial lability (van Os et al., 2003)—have been demonstrated to be common to the expression of psychotic phenotype and psychotic disorders. The emergence of some anomalous experiences could also be influenced by these factors and some studies from the field of anomalistic psychology have, for example, been conducted in “haunted” places in order to determine precisely which environmental features favor hallucinatory processes (Tandy and Street, 2000). These researches have notably demonstrated the impact of low frequency magnetic fields (Persinger, 2001) or infrasound (French et al., 2008a) on the emergence of hallucinatory processes in the general population and a “haunted” experimental room has been created in order to try to replicate these phenomena in controlled conditions (French et al., 2008a).

From a more emotional and dynamic point of view, these hallucinatory processes would be tied to a lack of integration of the emotional experiences, which would lead in turn to hallucinations. Reality monitoring could be influenced by emotional dynamics and meta-cognitive beliefs. Thus, when thoughts do not “fit” with our own system of beliefs and representations—for example, aggressive thoughts against a relative—we will tend to “externalize” these thoughts (Laroi and Linden, 2005) in order to avoid cognitive dissonance (Festinger, 1957). According to this theory, negative emotions are “projected” toward the exterior and are articulated, under some conditions, with hallucinatory processes (Laroi et al., 2005). Interestingly, this cognitive model of hallucinations seems to be aligned in part with psychoanalytical models and especially with the concept of “projective identification” which was defined by Klein (1946) as a process that “derives from anal and urethral impulses and implies expelling dangerous substances (excrements) out of the self onto the mother” (p. 99). Klein (1946) has underlined in particular the importance of this process during the “schizo-paranoid position,” in which the child projects the bad parts of the self and aggressive drive onto the mother in an indistinct manner. Psychoanalysts have since insisted on the fact that hallucinatory and perceptive fields are a consequence of subtle inter-relationships between intrapsychic thinking and their “projection” into the other. Psychotic patients would regress to the schizo-paranoid position, which in turn leads to a cleavage of reality, where the bad parts of the psyche are projected onto the environment and come back in an external manner.

Bion (1962) has further investigated these theories by showing how certain patients are terrorized by “bizarre objects” loaded with persecutory hostility. He attempted more generally to refine the concept of projective identification, arguing that it belongs to a global non-pathological process called “normal projective identification,” in which the psyche needs to project some part of itself in order to be integrated. In Bion's view, when some of the first mental constructions—called “Beta elements”—are not contained by the mother, they will produce the “bizarre objects” previously mentioned. Current analytical research has since developed this conceptual paradigm in order to understand the subtle relationship between intrapsychic and intersubjective processes (Roussillon et al., 2007). From this point of view, cognitive neurosciences would describe the neurobiological aspects of these processes, whereas psychoanalysts would underline their emotional and intersubjective dimensions.

Clinical practice with people reporting anomalous experiences frequently implicates this subtle play between intrapsychic content, hallucinatory processes, and projection. This idea is illustrated in the following clinical case:

Over the last month, Catherine has experienced strange and scary phenomena, like nocturnal “apparitions” and inexplicable noises, accompanied by a feeling of a presence in her house. Interestingly, she is not the only one in her house who has witnessed these phenomena, and other people around her, especially her husband, as well as her housekeeper, also report them. During the first interview, Catherine seems in a lot of anguish and she fears that she is “going crazy.” However, she does not have any antecedents of psychological issues and lives a “normal” life with her husband and her seven-year-old son. When Catherine is asked to describe what she reports as nocturnal apparitions, she describes a “small boy dressed in red whose face remains unseen.” She also explains that the inexplicable noises come essentially from the first level of her house. These noises can take the form of footsteps or something banging inside the walls. Catherine thinks it may be the spirit of a deceased person “stuck between two worlds” and she is scared that the haunting may be a premonition that something bad will happen to her son.

During several interviews, she provides details about the nature of the nocturnal apparitions. They are reoccurring and disturbing to the point that Catherine takes anxiolytic medication in order to sleep at night. It becomes progressively clear through Catherine's associations that she unconsciously fears that her son could be kidnapped. Indeed, before the first apparition, she explains having heard something like “the sound of a key” as if her son was leaving the house. Furthermore, the young boy that she sees wear red clothes and her son has recently lost some of his red clothes…After several weeks, Catherine has a dream in which she finally sees the face of the ghost boy, which is none other than that of her son, but “covered in scars.” She then associates this dream with her own terrible fear of darkness and death. She explains that, when she was a child, her mother would leave her in a closet in the dark for hours. She seems very moved when she expresses these feelings, and begins to elaborate links between the frightening apparitions and her own history as a child.

When Catherine tries to make sense of the noises, she explains that they probably come from “spirits of the dead” and she then evokes her deceased father, whose grave she “does not visit often enough.” She also refers to her mother who suffers from Alzheimer's disease, and Catherine says it is as if her mother was already dead to her. She seems to have a lot of pent up anger toward her mother, but she is unable to express her feelings because of her mother's state of health. Is she experiencing an unconscious guilt that could be an explanation of the beliefs of the return of spirits in her house? This hypothesis is supported by several associations that she makes between “the first level of the house”—where the noises supposedly appear—and “heaven.” Progressively, after several interviews over the span of 3 months, Catherine was able to gain insight about her own psychological dynamics and the nocturnal apparitions ceased. It seems that our exchanges concerning her own anxiety and her difficult grieving process helped Catherine draw parallels between her experience and her own psychological functioning, which favored the symbolization process and led to the disappearance of the hallucinations.

The experience described by Catherine could be interpreted at first sight as a brief psychotic episode associated with a paranoid interpretation concerning spirits. However, several aspects of her experience lead us to interpret what she reports as being more complex than just a traditional brief psychotic episode. First of all, Catherine does not seem to be delusional when she describes what happens to her, and she is able to express doubts about the reality of her strange experiences. Moreover, her interpretations in term of “spirits” come from associations during the session and can be considered as beliefs without being a firm delusional system, in comparison to what can be usually found with a delusional psychotic patient. Moreover, her experiences are a mix of hallucinatory processes and feelings of a presence that occurs most of the time during hypnagogic states and not during waking time. Finally, Catherine is not the only one to report the phenomena; these are also described by her husband, her son and their housekeeper, which is typical of “poltergeist” cases. This aspect raises the question of a possible illusion implicated in part in the experience reported by Catherine, rather than exclusively hallucinatory phenomena, in which there would be an objective source of noise that would be misinterpreted^4^. It also questions the influence of “affective contagion” (Wallon, 2000) inside the family as well as the group dynamic implicated in these kinds of experiences, which we won't analyse here. Considering our previous remarks about hallucinations, Catherine may be a good example of someone who could belong to the psychotic phenotype without the emergence of a clear psychotic disorder. It seems as if she was in an intermediary space, a kind of “buffer,” composed of elements of reality, illusion, projected elements in the environment and hallucinatory processes^5^.

Interviews with many patients reporting anomalous experiences, like Catherine, have led us to observe that these experiences usually appear during, or after, a strong emotional and negative experience (Haraldsson and Houtkooper, 1991; van Quekelberghe et al., 1991), for example an accident, a romantic break-up or the death of a loved one. We have proposed to call this specific reaction a “paranormal” or “anomalous” solution (Rabeyron et al., 2010), which most often blends hallucinatory processes and paranormal beliefs. There is frequently an inaugural paranormal experience, i.e., a first anomalous experience that leads to many others and that echoes an earlier trauma during childhood (Rabeyron et al., 2010). This link between negative life events, trauma, and anomalous experiences has also been confirmed using a quantitative approach showing that people report more traumas during childhood and negative life events in the year preceding the emergence of anomalous experiences (Rabeyron and Watt, 2010). This first and inaugural anomalous experience could be considered to be a specific coping strategy, which takes the form of an original reaction to negative life events. However, this strategy is rarely obvious to the patients, and most of the time they do not speak about the negative life events, or they do not spontaneously see a connection between the anomalous event and their personal life.

These observations concur with research on the influence of trauma on the emergence of a psychotic phenotype. For example, in a longitudinal study including 4000 adults from the general population, Janssen et al. (2004) showed that the risk of psychotic symptoms is 10 times greater after childhood abuse. van Os et al. (2009) also notes that traumatic or stressful experiences predict subclinical and transitory psychosis, which could overlap in part with the processes of anomalous experiences. Sommer et al. (2010) also found a high prevalence of trauma—especially emotional and sexual abuse—during interviews with patients suffering from auditory hallucinations, with the main distinction between the clinical and non-clinical populations being the positive or negative nature of the hallucinations. Thus, the same risk factors could provoke psychosis proneness and psychotic disorders, which suggests a common etiology. If there is a link between trauma and hallucinatory processes, it is also important to better understand if some attenuated versions of hallucinations are signs of a future psychosis, and why some patients will develop a chronic psychosis whilst others will not (De Leede-Smith and Barkus, 2013). In this regard, Hanssen et al. (2005) found that only 8% of people with transitory symptoms of psychosis maintain these symptoms long-term and fall into clinical psychosis. From this point of view, anomalous experiences probably share characteristics with the etiology of hallucinatory processes previously mentioned, even if their phenomenology cannot be reduced to them (Cardeña et al., 2014).

Beyond the influence of trauma on their emergence, another specificity of anomalous experiences is their frequent relationship with cultural and anthropological elements. As already mentioned, patients reporting anomalous experiences usually avoid psychiatric structures, being afraid that clinicians could “psychopathologize” their experiences and consider them to be pathological hallucinations. Consequently, many people look for cultural and anthropological material that helps them to make sense of their experience outside of a medical frame of explanation (Camus, 2002). The subject's environment would thus, play a key role in the arising of the paranormal solution. For example, even in western societies, people who witness apparitions have access to a large array of representations, beliefs, and alternative therapies (exorcists, ghost hunters, etc.; Favret-Saada, 1977) that help them to avoid taking into account their own psychological functioning. From this point of view, anomalous experiences seem to be a kind of “cultural buffer,” an intermediary cultural space where these borderline experiences find cultural material that helps people to make sense of these strange experiences.

## Anomalous experiences, altered states of consciousness, and structural analysis

Another approach to anomalous experiences, which is both complementary to and intertwined with hallucinatory processes, concerns their ties with altered states of consciousness, which include phenomena like sleep states and hypnotic processes. Many of these experiences—especially apparitions, psi perceptions, OBE and abductions—indeed occur when the person is falling asleep or waking up as seen in the case of Catherine. A first approach involves considering these experiences as a consequence of hypnagogic hallucinations. This type of hallucination may affect 37% of the population (Ohayon et al., 1996) and is often associated with sleep paralysis, i.e., the inability to move, accompanied by the impression of compression of one's chest. Twenty-five to thirty percentage of the population report having experienced sleep paralysis (Cheyne, 2003), and three phenomenological classes of experiences related to sleep states are usually described: vestibular motor hallucinations, intruders and succubus (Cheyne et al., 1999). Vestibular motor hallucinations are close to the phenomenology of OBE, while Intruder experiences include feelings of a presence that is frequently associated with visual, auditory and kinesthetic hallucinations. Succubus experiences are characterized by a difficulty in breathing, a suffocating feeling, an oppressive sensation in the chest area, and a feeling of an imminent risk of death. From a neurocognitive perspective, the occurrence of such phenomena could be the consequence of a faulty transition between rapid eye movement sleep and a waking state. EEG studies suggest that during sleep paralysis people are thus conscious even if they are in REM sleep (Cheyne et al., 1999). This specific state of consciousness may also induce hypervigilance, which in turn makes the subject more sensitive to environmental stimuli.

Whilst some people consider these experiences to be pleasant and are simply curious about their nature, others are tempted to interpret them using a paranormal framework. Some people believe that a diabolic or an invisible force is attacking them during their sleep. In some cases, simply informing and reassuring the subject about the physiological and neurobiological sources of their experience is sufficient. However, in other cases, these experiences are repetitive, distressing and linked to a more global intrapsychic dynamic. Abductions are an especially instructive example of anomalous experiences associated with sleep disorder during which people believe they have been abducted by aliens (Hough and Rogers, 2007). In some cases, the “memory” of the abduction is recovered during hypnosis sessions several years later, and patients often report that they have been the victim of a surgical intervention or a rape committed by aliens. They are usually very disturbed by this realistic experience and find it difficult to talk about. In some cases, the experience even leads to post-traumatic stress disorder and different kinds of psychosomatic symptoms (McNally et al., 2004; McNally and Clancy, 2005; French et al., 2008b).

Abduction experiences are particularly useful in allowing us to deepen our previous thinking concerning the nature of anomalous experiences, hallucinatory processes and their relationship with altered states of consciousness. We have seen so far how epidemiological and psychiatric studies shed light on the high frequency of hallucinatory experiences in the general population, as well as on the impact of trauma and environmental factors on the emergence of seemingly psychotic experiences. However, although these experiences look the same on the surface, they could correspond to different kinds of underlying processes. We have discussed very briefly Kleinian and Bionian's theories in which hallucinations are interpreted as being the consequence of projective phenomena in the environment. The structural model developed by Lacan (1932) proposes another perspective that improves our understanding of the nature of these experiences. From a Lacanian point of view, if abductions appear as a psychotic disorder (involving hallucinations of aliens and a belief in their existence)—and they will be sometimes considered in this way by clinicians—this would actually correspond to a neurotic structure. This is argued by Maleval and Charraud (2003) following the theories of Lacan; according to these authors, abductions would correspond to the acting out of a neurotic fantasy associated with elements from the cultural environment. More precisely, the abductee would be playing out a scene of seduction where the abducted body becomes another's object of desire.

In order to understand this distinction, it is necessary to say a few words on the Lacanian model of psychosis (Lacan, 1981). We can only partially describe the model here, given its complexity, but a brief description is useful in a more global manner to help us understand the processes of anomalous experiences and their relationship with altered states of consciousness. Thus, a distinction is usually made between the “return of the repressed,” as described in Freud's definition of neurosis (Freud and Breuer, 1895), and Lacan's definition of “psychotic foreclosure” (*forclusion*)(Lacan, 1981)^6^. In neurosis, repression processes concern the way in which repressed elements try to return or resurface in consciousness in a disguised manner (for example, in the forms of dreams or lapses). An illustration of this kind of neurotic process is given by Freud (1923) in *A Seventeenth-Century Demonological Neurosis*. Freud analyzed the evil visions of the Bavarian painter Christophe Haitzmann in reaction to a mourning situation (the death of his father) and the need to find a more comfortable situation from a material point of view (this occurred when he finally took holy orders). Freud interpreted Haitzmann's hallucinations as being neurotic, and proposed that they could be understood with the same logic as used to interpret dreams (Freud, 1900).

In contrast, in the psychotic structure analyzed by Lacan (1981), in continuation to Freud's intuitions about psychosis (1911), the symbolization process is attacked in itself (Freud, 1911). The “Name-of-the-father” (*Nom-du-Père*) (a fundamental signifier of symbolic castration) is never registered in the subject's symbolic universe (the forecloser of the “Name-of-the-father”) and produces a hole in the symbolization process. Thus, the subject is facing a collapse of the signifying chain and is “unable to signify aspects of his existence along the axes of metonymy and metaphor” (Redmond, 2013, p. 2). Psychic elements are thus foreclosed from the symbolic order and these elements return in the “real” (*réel*) hallucinatory reality of the subject. The psychotic person is then facing an “overflow of real” and elementary phenomena (e.g., strange intuitions, short and enigmatic hallucinations, intrusion of the signifier) as a consequence of the foreclosure (*forclusion*) of the Name-of-the-Father. He is facing the terrifying abyss of the foreclosure and has to manage to “bear” the contact with the real. The psychotic delusion will aim to “fill in” the hole in the symbolic order. Consequently, the psychotic structure, but also the psychotic transference, will be specific, as well as the psychotherapeutic approach of the patient. From this point of view, Lacan proposes that he be the “scribe” or the “secretary of the insane” (*le secrétaire de l'aliéné*) in order to help him to limit his *jouissance*. It is necessary to be in the position of a “witness” in order to help the patient to make sense of his history without interpreting what he says. In this way, the aim of the clinician is to “put the excess of real in the network of signifiers” (Brémaud, 2005, p. 697) in order to support and accompany the patient in the construction of what Lacan calls “suppletions” (*suppléances*).

Thus, even if the ability of the psyche to hallucinate does exist both in neurosis and psychosis, the contents and the form of what is perceived, as well as the underlying processes, are different. Psychosis and neurosis can both produce hallucinatory phenomena but the “neurotic hallucinations will be the consequence of the return of the repressed and are open to interpretation, but not the psychotic hallucinations produce by the mental automatism” (Maleval, 2014, p. 9). With a patient reporting anomalous experiences, the clinician will try in the same manner to understand, in this regard, the structure of the patient by “exploring the type of voice, the type of message, the interpretation of these messages, the previous experiences, the coping strategies, the emerging context, and the potential links with the history of the subject” (Maleval, 2014, p. 9).

Interestingly, these diagnostic debates relating to hallucinatory and anomalous experiences were described at the end of the nineteenth century by Pierre Janet, in his use of the term “extraordinary neurosis” (*névrose extraordinaire*) to describe the mental functioning of mediums (Evrard, 2013b). For Janet, this kind of neurosis involves patients with neurotic or hysterical structures showing clinical manifestations usually linked to psychosis, such as hallucinations and delusions. Maleval also pursued a distinction between the “psychotic delusion” (*délire psychotique*) and the “neurotic delirium” (*délirirum névrotique*) (Maleval, 1981). The psychotic delusion deals with the forclusion of the Name-of-the-Father, while the neurotic delirium concerns “the fantasmatic projections focused on the return of the repressed imaginary.” Confirming Janet's intuitions, and continuing the structural analyses of Lacan (1981), Spriet (2006) compared mediums' hallucinations with psychotic hallucinations, and found that they were different in several ways. Spriet came to the conclusion that a medium's experience is not psychotic but is rather the resurfacing of repressed content belonging to neurotic processes. For example, a psychotic patient might have a spontaneous impression that they received a call “from God,” while a medium will make a call “to God.”

In the field of anomalous experiences, Evrard (2014, pp. 286–287) has proposed to use this kind of criteria in order to make a clear distinction between an “anomalous experience of the neurotic” and an “anomalous experience of the psychotic,” depending on the type of: neologism, belief, dissociation, automatism, potential voices, address of the experience, hypnotizability, influence of the environment, anxiety, projection, and evolution of the potential delusion. Thus, some hallucinatory processes might appear both during “extraordinary neurosis” (a clinical expression of neurosis with hallucinations) and “extraordinary psychosis” (which corresponds to a clinical expression of psychosis; Evrard, 2013b, 2014). A clinical case of abduction will illustrate these theoretical propositions:

Beatrice is a 30-year-old woman who describes several different anomalous experiences. They started when she was a child by the vision of a “blue light” and a “black silhouette” bending on her bed. Her childhood is described more generally as an unpleasant one, with an alcoholic and violent father and a depressive mother who finally divorced. When she was a teenager, she was sexually abused several times by a close relative leading to an abortion she was not allowed to speak about in her family. Beatrice will develop, in adulthood, psi experiences (especially precognitive dreams), out of body experiences and apparitions, notably of a “white and vaporous shape” leading her to a profound feeling of relief after seeing it. But the main sources of suffering for Beatrice are several “visits” of what she supposed could be aliens, even if she is not really sure about it. During the first of these visits, a few years ago, she was on her bed and suddenly felt that she was paralyzed. She saw a “blue light” (the same one that she saw during childhood) and felt there was “something” close to her. When she turned toward it, she saw an “alien” taking her hand with “scratchy skin” and “black and empty black eyes.” She felt powerless and “entirely drained” before feeling a very intense pain in her shoulder. Then, the being disappeared, she cried and woke up her husband. Another visit happened a few months ago. She woke up in the night seeing this time a “naked body” whose gender or face she could not see. She felt that this “thing” went behind her and that she was suddenly paralyzed on her bed. She was “forced” to turn toward a “white being, with “almond eyes” that finally disappeared, leaving Beatrice in a very shocked state.

In the same manner as in the demonological neurosis analyzed by Freud in 1923, the phenomena experienced by Beatrice are labile and evolutive. They happen in a hypnoid state, concern essentially sexual and corporal aspects, and are not associated with a firm and definitive belief. Moreover, these elements appear in adulthood after childhood traumatic experiences. Like Beatrice, many people who report abduction experiences have indeed suffered from sexual abuse, and they frequently report sleep paralysis and hypnagogic hallucinations (McNally and Clancy, 2005). Thus, the abductions cases appear as a type of “crepuscural hysteria” (*hystérie crépusculaire*) as proposed by Maleval (1981)—an extreme form of neurosis that corresponds to the extraordinary neurosis of Janet—in which traumatic elements return in the psyche in a disguised manner. These elements are cleaved and “frozen” in the psyche, and the reappearance of this content is signaled by the return, in a hallucinatory form, of the traumatic experience^7^. Memories could be involuntarily retrieved by stimuli associated with the trauma, and some hallucinations may be the expression of such traumatic memories (Hardy et al., 2005). In abduction cases, this holds especially true for kinesthetic hallucinations, which may be a return of cleaved memories of sexual assault. There would be in both psychosis and neurosis a “hole” in the psychic reality, but its consequences on the psychic functioning and their hallucinatory expression will depend on the structure of the subject (Le Maléfan, 2001, 2014). In particular, as described by Maleval (1981): “the hysteric do not manage to accept her sexual body, while the psychotic has difficulties with the language (…) The psychotic is looking for an internal solution to the enigma of its being; the hysteric ask to Others the solution to her problems” (p. 112).

The return of traumatic elements will thus emerge sometimes in the intermediate area of anomalous experiences. This relationship between trauma and anomalous experiences can be traced back to a small study reported by Wilson and Barber (1983), which found a higher frequency of childhood traumas amongst a group of people with fantasy prone personalities. This result has been confirmed by further research on anomalous experiences, trauma and dissociation (Ross and Joshi, 1992). Irwin (1994) replicated these results with individuals whose parents were alcoholics, and this study led him to develop the “Childhood Factors Model,” in which anomalous experiences are the consequence of childhood trauma leading both to fantasy proneness (an escape thanks to imagination) and a strong need for interpersonal control (as a defensive coping mechanism).

The traumatic elements will lead during adulthood to the emergence of altered states of consciousness. These states of consciousness may emerge close to sleep as already mentioned, but they can also arise during broad daylight. From this point of view, current research about hypnotic states from cognitive neurosciences is helpful in order to help us better understand the processes implicated in these experiences. This implies another level of complexity because it is necessary to determine the neurobiological areas associated with hallucinations depending on the state of consciousness. This topic was first studied back in the eighteenth century, notably by Franz Anton Mesmer, who coined the term “animal magnetism,” by Le Marquis de Puységur, with the idea of “magnetic somnambulism” and, later, by Jean-Martin Charcot's study of hypnosis at La Salpêtrière (Ellenberger, 1994; Méheust, 1999). These early approaches—also developed by Janet, who associated the extraordinary neurosis with particular states of consciousness—emphasized that a subject under hypnosis can experience mental imagery in a particularly vivid manner. Today, research using functional Magnetic Resonance Imaging (fMRI) shows indeed that the cerebral zones activated under hypnosis are close to those activated when the person carries out the task in reality (Oakley and Halligan, 2013). Hypnotic states not only allow a person to “relive” events or to imagine new ones in a realistic manner, but also tend to modify certain visual, auditory, and kinesthetic aspects of the experience (Kosslyn et al., 2000). For example, whilst under hypnotic suggestion, some people can place their hand in scalding hot water without any discomfort (Rainville et al., 1999). Moreover, clinical observations not only show how easily some people can switch from one conscious state to another, but also how much one's psychological reality evolves and changes depending on these consciousness states (Cardeña and Winkelman, 2011). Many anomalous experiences arise in these “strange states of consciousness” (Valla and Pélicier, 1992) which could explain in part their realistic aspect, as is illustrated by the following case:

Helen had a very good friend who used to be her boyfriend a long time ago. He became sick and recently died. A few days after his death, she is walking down the street thinking that he could have lived a little bit longer if he had taken better care of his health. She starts feeling angry and having hostile thoughts toward him. Then, all of a sudden, she finds herself in “a completely different dimension” that she has trouble putting into words. She feels as if she is filled with “clarity, infinite knowledge, and an overwhelming love.” Then, she has a timeless experience of being able to hear and communicate with her deceased friend. Unable to explain this strong and emotional experience, she supposes that she has entered into a direct communication with another world, which she links with several coincidences that occurred after this experience. Subsequently, she also develops a high level of creativity and a baffling ability to reproduce the paintings of famous artists.

During the several interviews we had together, we worked on drawing lines between this experience and her personal history. Helene was born prematurely, separated from her mother for several months after her birth, and was raised in part by her grandmother, as were most of the women from her lineage. Her mother also did not have her own father present when she grew up, and men from her side of the family were “always absent.” On her father's side, many children had been abandoned. Furthermore, Helen explains having been “terrified” of being abandoned at the moment of her friend's death. Since this experience, she has become very sensitive and cannot stand interpersonal “attacks” that occur around her. This palpable fragility seems tied to her early life experiences and her intergenerational heritage. These aspects of her story could have catalyzed her reaction to her friend's death and may have awoken a defensive reaction to a strong separation anxiety developed in childhood. Thus, even if Helen is structured enough to not break into depression or delusion, her fragilities may have led her to have this disturbing mystical experience.

In Helen's case, the experience occurred after the death of a loved one, in a context of grief, which is also frequent in anomalous experiences. Moreover, although her experience was short, it has produced long-term effects. The experience appears as a kind of reassurance against anxiety, especially separation anxiety. It seems then, that, like Helen, when some people face a negative life event, they unconsciously fall into an altered state of consciousness in order to avoid the negative impact of the event. In this state of consciousness, they experience visual and auditory hallucinations in a very realistic manner, similar to self-hypnotic states. The fluctuation of a person's hypnotizability according to the situation they are in has indeed been shown in other domains and, for example, people approaching the end of their lives are more easily hypnotized (Spiegel, 1985). When external reality becomes too difficult to live in and to accept, some people will become more sensitive to hypnosis and unconsciously enter into altered states of consciousness. Moreover, if we refer to our previous structural analysis, the case of Helene, as well as the cases of Beatrice and Catherine, belongs to a neurotic register, in reaction to a negative life event and a traumatic childhood. In Helene's case, the hallucinatory experience seems to correspond to the pleasure principle in reaction to the trauma: her experience allowed her to “retrieve” the lost relative in the same manner that the evil replaced the deceased father in the case described by Freud of a demonological neurosis.

## Anomalous experiences, hypersensitivity, and psychic permeability

If this structural analysis is useful for the purpose of proposing a distinction between the processes implicated in anomalous experiences, other psychoanalytical and complementary models also help to improve the intelligibility of these experiences. In particular, the altered states of consciousness and their links to hallucinatory processes seem to be enlightened by contemporary work about the concept of figurability (*Darstellbarkeit*) (the ability of the psyche to make the absent object present in images). Thus, Green (1993) has proposed a theory of hallucinatory and figurability processes which describes their link with the construction of mental processes. He supposes that we progressively develop an internal space of representation in encounters with the maternal environment. The mother has to be progressively negatively hallucinated—defined as “the non-perception of an object or of a perceptible psychical phenomenon” (Green, 2005, p. 216)—in order to let the child develop his/her own representation of her (Green, 1993). There will thus be a constant and complex inter-relation between negative hallucinations (the ability to not perceive the present object) and positive hallucinations (the ability to produce an image of the absent object). Anomalous experiences appear in this regard in the form of a “spillover” of this internal and negative hallucinatory frame, when something of the internal processes of the subject cannot be contained and these experiences may be considered to be “fluctuations” or “failures” of the hallucinatory system.

Botella and Botella (2005) have deepened Green's theories by analyzing the links between figurability processes and traumatic events. They define figurability as “a psychic property determined by a tendency toward convergence, the actualization of which triggers a process of binding all the constituents, all external and internal stimuli” (Botella and Botella, 2005, p. 12). They have analyzed, by means of clinical cases in analysis, how regression states during seances allow the patient to produce a hallucinatory figuration of a traumatic event, resulting in “accidents of thoughts” both for the analyst and the patient. Anomalous experiences could be considered from this point of view to be some of these “accidents of thought” happening outside the analytical setting. They are an attempt by the psyche to develop a figurative hallucination in a particular state of consciousness, which would give birth to an integrated representation of traumatic experiences. The emergence of this kind of hallucinatory processes is frequently associated with a pronounced sensitivity which is also present in most of the anomalous experiences. This strong sensitivity also helps us to better understand the strong influence of cultural elements on the subject. Another example will illustrate this hypersensitivity:

Palma describes the impression of being able to feel the emotions of others and has had several precise “flashes” and “intuitions” about relatives' lives. This sensitivity—shared with her mother and her grandmother—suddenly increased after a traumatic accident she had at work a few years ago. However, as a child, she already used to see “entities” and apparitions, which she still sees. She also reports frequent premonitory dreams. At her work, this sensitivity is especially intense and she frequently loses consciousness because of it. She explains that she is like a “sponge,” as if she could “feel” what others feel, which has made her coworkers progressively afraid of her. She has eventually become disliked and rejected, and some of her colleagues have started referring to her as the “witch,” while others have told her that she was a medium. She finally attended training with a healer and met a medium who confirmed her “mediumistic abilities.” More generally, Palma describes her childhood as being particularly insecure and violent, and interprets her sensitivity as a reaction to a life that was “made up of shocks” and an attempt to uphold close ties to others in order to anticipate their reactions.

This feeling of being like a “sponge” and having very “thin boundaries” is common amongst people who report anomalous experiences. This sensitivity was also present in the theories of Myers (1903) who coined the term “transliminality” in order to describe this psychological peculiarity. He described it as a tendency of certain psychological contents to easily pass through the barrier between the conscious and the unconscious mind. A transliminality scale has since been developed, followed by a revised version named the Rasch Revised Transliminality Scale (Lange et al., 2000). People who report anomalous experiences score higher on transliminality scales than those who do not have such experiences, and transliminality has also been correlated with trauma during childhood (Thalbourne, 2000; Thalbourne et al., 2003). The notion of mental boundaries has also been widely studied by Hartmann (Hartmann, 1991; Harrison et al., 2006) who distinguishes between several boundaries in the mind that can be either thicker (impermeable) or thinner (permeable). Thinner psychic permeability engenders “unexplained” interactions with the environment and increases porosity to primary processes, leading to feeling-related, empathetic and intuitive thoughts. These feelings are sometimes interpreted afterwards as a “sixth sense” or a “magic” link to other people. Psychic permeability also leads to a strong sensitivity to the thoughts of others, which could take the form of “flashes” and shared feelings.

Findings in neurosciences relating to mirror neurons, initiated by Rizzolatti et al. (1996), shed light on the concept of psychic permeability by underlining that certain types of neurons are activated either when we carry out an action or when we see someone else doing it. These neurons are differentially activated according to the task at hand (e.g., picking up a glass) and the associated intention (e.g., picking up a glass in order to drink water) (Iacoboni and Dapretto, 2006). Mirror neurons also play an important role in social cognition, leading to the “chameleon effect,” i.e., the unconscious tendency to automatically reproduce the emotional and psychological states of others (Chartrand and Bargh, 1999). These neurons also help us understand the development of empathy: we perceive the emotional states of others by activating the same emotional state in ourselves (Rizzolatti and Craighero, 2006). We are thus intimately tied to those around us, and these interpersonal processes are fundamental for our neurological and psychological functioning. Some studies suggest that patients suffering from autism could have a deficiency in the activation of this category of neurons (Dapretto et al., 2006; Honaga et al., 2010). Inversely, anomalous experiences may be the sign of a hyper-activation of these neurons. Instead of an activation deficiency, some people could have mirror neuron areas that are more easily aroused, which could take the form of the “hypersensitivity” previously evoked, as illustrated in the case of Kyra:

Kyra describes herself as a very sensitive person and reports several anomalous experiences. She first speaks about several OBEs, but she also frequently sees “entities” and sometimes practices magnetism. She often hears strange noises in her house, and she once felt paralyzed on her bed. This was a very disturbing experience during which she had the feeling that an entity was lying on her back. Since then, she has had the impression of having a “hole” in her back where entities can “stick” to her when she is in a “bad mood.” Despite these strange experiences, Kyra manages to work (in the field of art), and has a son who also sees the entities and who even draws some of them. One of the main characteristics of Kyra seems to be that she has very thin mental boundaries. For example, when someone is feeling bad around her in the subway, she feels a strong discomfort in her own body. Interestingly, she has a condition called Darier's disease in which “holes” can appear in her skin and can easily become infected, as if the thinness of Kyra's mental boundaries was also present in her own skin.

As seen in the cases of Kyra and Palma, a higher degree of sensitivity can lead to the feeling of being able to automatically detect the emotional states of others. They seem to be “contaminated” by other people's emotions and cannot tell the difference between them and their own feelings. In this regard, they could be described as having an easily aroused “same system,” which is described by Georgieff (2008) as a “process that allows the reproduction of cerebral and mental activities of an individual by those of another individual” (p. 365). This “same system” is associated with a “who system” that enables the individual to identify secondarily what is felt as belonging to him. When these two systems do not function normally, they engender what Georgieff calls “empathy pathologies,” which are close to what is found in anomalous experiences. This porosity between oneself and others seems to be frequently associated with strong suffering in childhood. Some anomalous experiences thus correspond to a form of “hyper-tuning” with the environment in reaction to early traumatic events. This hyper-tuning also produces a decrease in the boundaries with the perceptive environment and leads to an attenuation of the frontiers between internal representations and their expression in the perceptive field.

Psychoanalytical models, especially those derived from the Anglo-Saxon tradition, give a certain “thickness” to the understanding of this hypersensitivity associated with anomalous experiences, by connecting these observations to the way the psyche is constructed. In particular, Winnicott's (1958) and Bion's (1962) theories shed light on these processes by underlining the role of the child's environment and the primary mother-child relationship for the construction of the psyche. They suppose that the child alone is not able to integrate his emotional states and he needs to encounter an “Other” which will help him to integrate his affective experiences. The child then progressively constitutes his own psychic reality on the basis of these early relationships and these interactions become the foundation of the dynamic interaction of his own psychological processes. As summed up by Roussillon et al. (2007): “the reflexive loop keeps the trail of the intersubjective loop.” Bion (1967) also studied these processes with the notion of a “contact-barrier” that “separates mental phenomena into two groups, one of which performs the functions of consciousness and the other the functions of unconsciousness” (p. 22). The quality of this barrier is determined by early relationships, varies widely from one person to another and probably has an impact on the emergence of anomalous experiences. Anzieu (1974), poursuing the theories of Bion, suggested for his part the existence of the “Skin-Ego” (*Moi-Peau*) which is based on the idea that “psychic envelopes” are specifically formed according to a model of the functions of the skin. As illustrated by the case of Kyra, the foundation of the skin and the Ego seem to be closely entangled: “holes” in the skin could be associated with “holes” in the psyche and lead to the access of elements that would normally remain unconscious.

Federn (1956) had also analyzed this kind of hypersensitivity to the environnement with what he calls the “frontiers of the Ego.” He studied the “normal breath” of the Ego and the “feeling of the Ego,” which varies according to the different states of consciousness, and which he ties to empathy and telepathy (p. 257). Federn describes the constant fluctuation of mental boundaries, which leads, in some situations, to mysterious modifications in the perception of the Self that can take the form of anomalous experiences, especially mystical experiences. More specifically, he suggests that psychic permeability is “two-sided,” with a strong psychic permeability between conscious and unconscious processes, and also between the internal and external worlds. We propose that these processes are also “two-directional,” as they can lead to both perceptive (e.g., in psi experiences and OBE) and projective (e.g., in magnetism experiences) processes. The cognitive and affective functioning associated with these experiences then constitutes a disadvantage corresponding to difficulties in the psychological categorization between internal and external worlds, but also an advantage because they may then lead to a capacity for healthy adaptation and to the heightened expression of creativity.

This potential increase in psychic permeability will take different forms depending on the multiple forms of traumatic configuration at its origin. We have observed that in a number of clinical cases this kind of permeability seems to appear in patients suffering from pronounced narcissistic suffering, especially patients who seem to have been confronted during their early childhood with a mother going through a major depressive episode. Green proposed to name this phenomenon the “Complex of the dead mother” (Green, 1983). Here, patients suddenly confronted with a depressed mother experience a “failure of the experience of “individualizing separation,” where the young Ego, instead of constituting the container of subsequent investment after the separation, tries to retain the primary object and repetitively relive its loss, which induces at the level of the primary ego confounded with the object (…) a feeling of void characteristic of depression” (p. 248). This concept echoes what was described by a patient, Petra, who reported that her mother was suddenly and profoundly depressed during her early childhood. She later developed, after a breakup, strong and frequent psi perceptions involving her ex-girlfriend. In this kind of case there is not a clear trauma but rather a form of “mark” or “trail” let into the psyche by primitive experiences during early relationships. These “marks” would then take the form of a hypersensitivity with two main unconscious purposes. The first one would be to keep the link to the Other in a desperate manner, in order to retain or avoid the psychic separation with the primary object and to repair the damage caused by this early loss. From this point of view, psi experiences would be a way to maintain the feeling of being linked to the other by being able to guess his or her thoughts. The second function of this hypersensitivity usually appears after later mistreatment during childhood, and manifests itself as an attempt to be very sensitive to variations in the environment, and thus anticipate possible abuse.

## Anomalous experiences and extreme forms of symbolization

A short summary seems necessary before going further in our analysis concerning the links between anomalous experiences and symbolization processes. We have so far tackled how hallucinatory experiences are present in the general population, which have led psychiatric epidemiology to consider the existence of a psychotic phenotype widely distributed in the population. Anomalous experiences could also fit into this category of hallucinatory experiences, even if, as underlined notably by Le Maléfan (2014) “hallucinatory as well as delusion experiences and anomalous experiences do not overlay entirely” (p. 169). A number of factors would nevertheless be common between them, like environmental factors, the influence of traumas during childhood and probably some neurobiological mechanisms. Psychoanalytic approaches, especially the Lacanian structural model, propose more precisely that there may be a difference of *nature* rather than a difference of *degree* between the different forms of hallucinatory processes, depending on the neurotic or psychotic underlying structure (Evrard, 2014)^8^. This difference is also useful in helping us to understand the process of symbolization associated with anomalous experiences.

As illustrated by the five clinical cases proposed in this paper, if anomalous experiences look like psychotic experiences, most of the time they are likely to be associated with a neurotic structure from a psychoanalytical and structural point of view. More precisely, from what we can observe in the counseling service, four persons out of every five possess an underlying neurotic structure (Evrard, 2014). Thus, anomalous experiences relate most of the time to an extreme form of expression of neurosis, which confirms the analysis of Janet (Evrard, 2013b) and Maleval (1981). These experiences will emerge most of the time during altered states of consciousness and are linked to a pronounced psychic permeability, leading to a form of sensitivity to cultural material associated with the paranormal. Nevertheless, even if the processes of these experiences are better understood by means of the structural model, they cannot be entirely reduced to it. For example, a number of anomalous experiences appear as being “trans-nosographic” given their heterogeneity, their multiple context of emergence and the complexity of their processes. For example, NDEs or OBEs emerge in both neurotic and psychotic structures without a clear difference in the way they are described (Rabeyron and Caussie, in press). Thus, even if both psychiatric and structural points of view are relevant and improve our understanding of these experiences, they do not entirely explain the logic of the processes of anomalous experiences.

Contemporary models of symbolization based on the “metapsychology of processes” (Roussillon, 2001, 2015) offer a complementary point of view to the Lacanian approach, especially in the continuation of Bion's work. This model supposes in particular a stronger permeability between the different psychological processes belonging to neurosis and psychosis, and places more focus on narcissistic and self-identity suffering. We will not explain here the commonalities and differences between the Bionian and the Lacanian models^9^; our aim is rather to propose a complementary “vertex” of heuristic value in order to improve the intelligibility of anomalous experiences. The metapsychology of processes seems especially relevant in allowing us to understand the archaic and symboligenic aspects of primary forms of symbolization, and appears easier to integrate with cognitive neuroscience discoveries.

Thus, following most keenly the model of symbolization suggested by Bion (1962), several French psychoanalysts (working mainly with psychotic and borderline patients in the 1970s in France) have developed concepts that detail the pre-symbolic construction of the psyche, notably Anzieu's (1987) “formal signifiers” and Aulagnier's (1975) “pictograms.” These theories—which we cannot describe in detail here—analyze precisely the processes of transformation of the psyche and the early forms of subjective appropriation. They underline how the psyche produces its first forms of thoughts in close articulation with the body and the environment. Along the same line of thought, Roussillon (1999, 2015) has proposed more recently that there may be a distinction between primary and secondary symbolization processes. The first level of transformation (primary symbolization) concerns essentially non-verbal experiences, while the second level of transformation (secondary symbolization) deals especially with the verbal description of subjective experiences. Roussillon (2015) defines primary symbolization more precisely as “the process that moves from the “raw material” of experience, the perceptive mnemic trace (…) which carries the sensory-motor trade of the impact of the encounter of the subject with a still poorly differentiated, poorly identified object, confusing part of the subject and part of the object, to a possibility of scientification which is able to “become language,” able to be narrated to another subject, to thus be shared and recognized by another subject to in turn become assimilable into subjectivity” (p. 594). A deficit of integration of the subjective experience at the different levels of psychic transformation will lead to multiple forms of expression of human suffering.

From this perspective, hallucinatory processes, states of consciousness and psychic permeability can be considered to be three complementary aspects of the symbolization process implicated in anomalous experiences. They show how the psyche represents and transforms its past and current internal experience in extreme situations. The psyche can use these “cursors” in order to handle the subtle tuning between the internal and the external worlds, between the subject and its environment. Anomalous experiences appear from this point of view at the frontiers of the symbolization abilities of the psyche; they are attempts to explore new and extreme forms of psychic solutions. These experiences are also at the frontiers of subjectivity and they implicate a process of transformation that is a risk in itself because it can also overflow the capabilities of psychic transformation. They can thus have both positive and negative consequences, explaining the difficulty in categorizing these experiences from the point of view of psychopathology.

Cognitive neurosciences also explore how the brain gives birth to subjectivity and can improve our understanding of the emergence of anomalous experiences. Damasio (2012) and Edelman (1990) both describe how early stages of functioning lead progressively to the development of consciousness and subjectivity. Damasio (2012) proposes in particular a distinction between the protoself (neural patterns representative of the body's internal state), the core consciousness (the organism becoming aware of its own feelings) and the extended consciousness (higher levels of thought that implicate time and space, and lead to autobiographical memories). Edelman (1990) and Edelman et al. (2011) describes for his part a distinction between primary consciousness and a higher order of consciousness relying on a dynamic core (a pattern of neural activity leading to particular mental functioning), and the reentry (continual signaling from one brain region to another) of brain maps progressively selected by evolution. These models shed light on the fact that subjectivity is a consequence of several layers of consciousness, progressively developing from primary consciousness to higher orders of consciousness, and involving complex inter-relationships with the environment.

These models also underline the idea that primary consciousness and higher order consciousness are always interconnected. Earlier phases of development are still present in the functioning of the human mind, and anomalous experiences could be considered as a return or a regression to earlier modes of primary symbolization at a concrete presymbolic level of the psyche^10^. The process of transformation implicated in anomalous experiences concerns in particular primary symbolization processes, which lead to specific modes of integration and transformation of the subjective experience. They can be conceptualized as an extreme form of symbolization associated with primitive functioning of the psyche, where there is a lack of distinction between the self, the body and the environment. From this point of view, anomalous experiences give an original and specific insight into the functioning of the symbolization process. For example, these experiences frequently produce a feeling of interconnectedness (similar to that described by Helen), which can be understood as a reappearance of a feeling of primary communication when the Ego and the environment are not separated. Another example is provided by magnetism experiences, which can also be seen as a return to the feeling of the kind of “magical” interaction between the mother and her baby. They could be seen as a metaphor of the regression to early relationships in which the mother transforms the feelings and “heals” her baby, in order to increase symbolization processes. Thus, anomalous experiences could be a kind of “vestige” of these primary forms of intersubjective links.

These processes are often present during crisis situations and imply a psychological survival process, which acts as a kind of “shortcut,” and whose main function is to contain overwhelming emotions in order to protect the psyche from a potential collapse. For example, the ability to perceive oneself “outside” one's body may have a protective function during situations of psychological or physical danger (Greyson, 2007; Rabeyron, in press). This defensive process probably relies on a sudden increase of permeability in the brain which modifies psychological associativity (Edelman, 1990) associated with specific neurobiological functioning. Some research suggests in particular that anomalous experiences are linked to an increase in temporal lobe ability, a reduction of cognitive inhibition and an increase in communication between the higher and lower parts of the brain, especially between the cortex and the limbic system (Simmonds-Moore, 2010). This increase could also take different forms depending on the type of anomalous experience, considering that such a mechanism represents a sort of “rescue system” in which the normal laws of cognitive associativity are modified when the subject is confronted with a strong emotional disturbance. This mechanism is probably crucial in the emergence of anomalous experiences, and would correspond to a coping strategy comparable to the “strategy of the reed,” in which the psyche chooses to bend instead of breaking. However, some people will not be able to integrate this specific anomalous pattern, which would then result in a psychological collapse, as can be seen, for example, after some mystical experiences (Chouvier, 2006). Thus, the same processes that are seen at the origins of the construction of the psyche suddenly reappear in an original manner when metabolization processes are overwhelmed, and the same particular functioning of the psyche is to be found both in primary relationships and anomalous experiences.

## Concluding remarks

Anomalous experiences occur when a person is confronted with an experience that does not fit with his usual conception of reality, an “extraordinary” interaction with the environment that can produce both a positive and a negative impact on mental health. We have proposed several ways of considering and explaining their phenomenology, relying on research about hallucinatory processes, altered states of consciousness, and psychic permeability, which are the main specificities of an “anomaly prone personality” (Simmonds-Moore, 2010). In order to better understand the processes linked to these experiences, a psychoanalytical and Lacanian structural approach has been useful in allowing us to recognize the particularity of these experiences depending on the structure in which they emerge. We have then proposed an analysis relying on the primary forms of symbolization in order to study the symboligenic aspects of anomalous experiences.

In additition to these psychoanalytical models, the double stance proposed by crossing the psychoanalytical approaches and cognitive models is essential in order to shed light on the specificity of anomalous experiences. More precisely, the differences in the current psychoanalytical models could benefit from knowledge of cognitive neurosciences, and the position at the “frontiers” of anomalous experiences could be part of this dialogue. For example, in the same manner as we are able to better understand the different areas implicated in dreams by crossing psychoanalysis and neuroimagery (Kaplan-Solms and Solms, 2002; Ruby, 2011), a similar approach could be progressively developed in the field of anomalous experiences. One of the best examples is provided by research concerning OBE, which allows us to draw bridges between subjective experiences and neurological functioning, in order to improve our knowledge of embodiment processes. A number of studies have recently been conducted on other anomalous experiences, especially with regards to mystical experiences (Beauregard and Paquette, 2006), and NDEs (Thonnard et al., 2013). If cognitive neurosciences help us to better understand the neurological specificity of these experiences, psychoanalysis facilitates our understanding of their impact on subjectivity.

The use of both psychoanalysis and cognitive neurosciences also allows us to avoid “mutilating” these experiences, by taking into account the different levels of reality they imply. Indeed, a trap of which we must steer clear would be to assume that anomalous experiences are of a purely neurological nature, all the while ignoring the emotional context of their emergence and the associated meaning of the transformation and metabolization processes they imply. A second trap would be to focus only on the meaning and the phenomenology of these experiences, while neglecting to consider their physiological and neurological correlates and their more global neurological functions. Future studies on this topic should continue to bring these aspects together, as in the more global projects developed by neuropsychoanalysts (Solms and Turnbull, 2011), in order to develop an integrative model of anomalous experiences.

This project could also help to better inform clinicians on how to help people who have difficulties in coping with anomalous experiences. As proposed by a recent paper in the *American Journal of Psychiatry* (Lomax et al., 2011) describing the clinical case of a patient in psychodynamic therapy reporting several anomalous experiences, it is important “to encourage clinicians to be open to their patients' descriptions of anomalous experiences and to work with patients to construct meaning of these experiences that will promote health, positive coping, and growth” (p. 12). The clinician has to be able to recognize these experiences which could be “fundamentally about creating (or revealing) meaning, narrative, and story to an individual in some difficult or even traumatic situation” (Lomax et al., 2011, p. 15). In particular, it is important that clinicians help their patients to integrate and make sense of these unusual and disturbing experiences. This can be accomplished by having an open attitude, and by avoiding adverse responses such as fear, rejection, and fascination. There is indeed a kind of “address” of the anomalous experience as a trail of the aborted transformation process, and the experience demands to be shared for the same reason. Research in these directions could then lead to original theoretical and practical implications in further classical domains—for example, non-pathological hallucinations and their expression in the general population, or extreme forms of symbolization—which may be useful for both psychoanalysis and neurosciences.

### Conflict of interest statement

The authors declare that the research was conducted in the absence of any commercial or financial relationships that could be construed as a potential conflict of interest.

## Footnotes

^1^Another recent example concerns, on a more experimental level, studies based on psi perceptions which have contributed to an ongoing debate about methodology and replicability in psychology and medical research (Bem, 2011; Wagenmakers et al., 2011; Rabeyron, 2014).

^2^Before being interviewed, the patients were asked for permission that the clinical material collected during the interviews could be used for scientific publication. All cases were anonymized and transformed in order to make the patients unrecognizable.

^3^The Center for Information, Research and Counseling about Exceptional Experiences (CIRCEE). Most of our patients are referred by clinical psychologists and psychiatrists, but we are sometimes contacted through our website (www.circee.org). On average, 300 people contact us per year. There are several other counseling services that specialize in these experiences in Europe, for example the Institute for Frontier Areas of Psychology and Mental Health (IGPP) in Freiburg, Germany (Fach et al., 2013).

^4^A classical and surprising example is the noise made by an owl in an attic, which sounds like the noise made by human footsteps.

^5^Later developments in the paper will propose a more precise interpretation of the nature of this kind of hallucinatory experiences.

^6^The Lacanian forclusion is close to the verwerfung in Freud's writings.

^7^In this sense, abductions would correspond to a real traumatic event, but from another time, in the same way that “primitive agony” during childhood later elicits a “fear of breakdown” (Winnicott, 1974; Roussillon, 1999).

^8^It would be interesting, from this point of view, to question the possibility to study such a difference of structure thanks to the current cognitive neurosciences tools.

^9^We recommand to the reader interested in the differences between the Lacanian and Bionian model the written retranscriptions of Pierre-Henri Castel's seminars (2006-2007) untitled ≪ Lacan/Bion: qu'est-ce que l'appareil psychique? ≫ available here: http://pierrehenri.castel.free.fr/

^10^We can't describe here in detail the complex links between symbolization processes in neurosciences and psychoanalysis. The reader can find more information on this topic in Rabeyron (in press).
